# Neighborhood Disadvantage, Greenness, and Population Density as Predictors of Breastfeeding Practices: A Population Cohort Study from Finland

**DOI:** 10.1093/jn/nxac069

**Published:** 2022-03-24

**Authors:** Laura Galante, Mirkka Lahdenperä, Samuli Rautava, Jaana Pentti, Helena Ollila, Saija Tarro, Jussi Vahtera, Carlos Gonzales-Inca, Mika Kivimäki, Virpi Lummaa, Hanna Lagström

**Affiliations:** Department of Biology, University of Turku, Turku, Finland; Department of Public Health, University of Turku and Turku University Hospital, Turku, Finland; Department of Biology, University of Turku, Turku, Finland; Department of Public Health, University of Turku and Turku University Hospital, Turku, Finland; Centre for Population Health Research, University of Turku, Turku, Finland; Department of Pediatrics, University of Turku, Turku, Finland; Department of Pediatrics, University of Helsinki, Helsinki, Finland; Children's Hospital, Helsinki University Hospital, Helsinki, Finland; Department of Public Health, University of Turku and Turku University Hospital, Turku, Finland; Clinicum, Faculty of Medicine, University of Helsinki, Helsinki, Finland; Clinical Research Centre, Turku University Hospital, Turku, Finland; Department of Public Health, University of Turku and Turku University Hospital, Turku, Finland; Centre for Population Health Research, University of Turku, Turku, Finland; Department of Public Health, University of Turku and Turku University Hospital, Turku, Finland; Centre for Population Health Research, University of Turku, Turku, Finland; Department of Geography and Geology, University of Turku, Turku, Finland; Clinicum, Faculty of Medicine, University of Helsinki, Helsinki, Finland; Helsinki Institute of Life Science, University of Helsinki, Helsinki, Finland; Department of Epidemiology and Public Health, University College London, London, United Kingdom; Department of Biology, University of Turku, Turku, Finland; Department of Public Health, University of Turku and Turku University Hospital, Turku, Finland; Centre for Population Health Research, University of Turku, Turku, Finland

**Keywords:** human milk, health inequalities, early life nutrition, environmental health, social disadvantage, nursing behavior, lactation, mother-infant dyad

## Abstract

**Background:**

Many environmental factors are known to hinder breastfeeding, yet the role of the family living environment in this regard is still poorly understood.

**Objectives:**

We used data from a large cohort to identify associations between neighborhood characteristics and breastfeeding behavior.

**Methods:**

Our observational study included 11,038 children (0–2 years) from the Southwest Finland Birth Cohort. Participant information was obtained from the Medical Birth Register and municipal follow-up clinics. Neighborhood socioeconomic disadvantage, greenness, and population density were measured for a period of 5 years prior to childbirth within the residential neighborhood on a 250 × 250-m grid. Any breastfeeding and breastfeeding at 6 months were the primary outcomes. Binary logistic regression models were adjusted for maternal health and socioeconomic factors.

**Results:**

Adjusted analyses suggest that mothers living in less populated areas were less likely to display any breastfeeding (OR: 0.46; 95% CI: 0.36, 0.59) and breastfeeding at 6 months (OR: 0.37; 95% CI: 0.34, 0.40). Mothers living in highly disadvantaged neighborhoods were less likely to display any breastfeeding if the neighborhood was less populated (OR: 0.54; 95% CI: 0.30, 0.95) but more likely to breastfeed at 6 months if the neighborhood was highly populated (OR: 3.74; 95% CI: 1.92, 7.29). Low greenness was associated with higher likelihood of any breastfeeding (OR: 3.82; 95% CI: 1.53, 9.55) and breastfeeding at 6 months (OR: 4.41; 95% CI: 3.44, 5).

**Conclusions:**

Our results suggest that neighborhood characteristics are associated with breastfeeding behavior in Finland. Unravelling breastfeeding decisions linked to the living environment could help identify interventions that will allow the appropriate support for all mothers and infants across different environmental challenges.

## Introduction

Breastfeeding is regarded as the gold standard for infant nutrition. Maternal milk provides the optimal building blocks for postnatal growth and development (1) and confers health benefits for both short- and long-term health outcomes (2), including improved neurodevelopment (3) and a generalized reduction in the risk of obesity (4) and infectious diseases (5). By potentially improving intelligence quotient, school attendance, and lowering the risk of noncommunicable diseases, it is likely that breastfeeding could help alleviate public health problems worldwide, if all infants between 0 and 23 mo were breastfed according to the WHO and UNICEF guidelines (6). Yet, in high-income countries more than 1 in 5 infants are never breastfed (7), and the rates of prolonged breastfeeding after the first 6 months and up to 23 months are the lowest for developed countries. In Finland, for example, recent evidence suggests that only 60% of all infants might be breastfed (partially) at 12 months, although rates have been increasing since 2010 (8).

Breastfeeding challenges, which impact on breastfeeding guidelines adherence, have been hypothesized to be rooted in the society and environment in which mothers live (9). Factors affecting breastfeeding behavior include a family's socioeconomic status (SES), education, income, and lifestyle (10); neighborhood SES; and population density/urbanization (11, 12). All these could be related to adoption of suboptimal breastfeeding practices (13, 14), altered maternal milk composition (15), and suboptimal growth trajectories in the infant beyond the first year of life (16). Yet, associations between the neighborhood SES and breastfeeding practices have only been explored in a few instances, with authors reporting that low neighborhood SES is associated with poor breastfeeding practices, at least in some groups of mothers (14, 17, 18). The link between population density and breastfeeding behavior has been studied in developing countries with conflicting results (13, 19), and fewer data are available from Western societies (20, 21). The presence of green areas in the neighborhood has been associated with health outcomes throughout the lifespan, and greater greenness has been linked to increased well-being (22). However, whether the presence of green spaces in the residential neighborhoods is associated with breastfeeding practices remains, to our knowledge, unknown. Lastly, the significance of the duration or timing of exposure to the above-mentioned neighborhood characteristics and breastfeeding behavior is also at present unknown.

Accordingly, in the present study we investigated the association between 5-year cumulative exposure to neighborhood socioeconomic disadvantage, greenness, and population density, and breastfeeding practices in an unselected population-based cohort of all children born in southwest Finland in a 3-year period. We hypothesized that high neighborhood disadvantage is linked with suboptimal breastfeeding practices, whereas high greenness and lower population density would be linked to a breastfeeding behavior more compliant with the current guidelines.

## Methods

### Study population

The present study is based on data collected within the Southwest Finland Birth Cohort, a longitudinal 3-y birth cohort consisting of all children born between January 1, 2008 and December 31, 2010 (*n* = 14,946) in the Hospital District of Southwest Finland and their mothers (*n =* 13,436). At the time of the study, the district included 2 hospitals (23). Consequently, the study cohort consisted of all children born in the geographical area during the 3-year period. For the purpose of the present study only the first child born from each mother during this time period and for which breastfeeding information was collected was included (*n =* 11,038). Additionally mother-infant dyads with missing information on neighborhood socioeconomic disadvantage (*n =* 472), missing information on population density (*n =* 85), and missing information on greenness (*n =* 86) were excluded from models that included these variables (**Supplemental Figure 1**). The study was approved by the Ethics Committee of the Finnish Institute for Health and Welfare. The legal basis for processing of personal data is public interest and scientific research [EU General Data Protection Regulation 2016/679 (GDPR), Article 6(1)(e) and Article 9(2)(j); Data Protection Act, Sections 4 and 6].

### Pre- and perinatal characteristics

Pre- and perinatal characteristics including child sex, maternal age at birth, number of previous births, marital status, maternal occupational status, smoking during pregnancy, maternal prepregnancy BMI, maternal chronic and pregnancy diagnoses, mode of delivery, and gestational age were extracted from the national register on parturients, deliveries, and births maintained by the Finnish Institute for Health and Welfare. Maternal chronic and pregnancy diagnoses based on International Classification of Diseases, 10th Revision codes included cancer, diseases related to the nervous system, mental and behavioral disorders, cardiovascular diseases, respiratory diseases, digestive tract diseases, diseases of the musculoskeletal system, diseases of the genitourinary system, hypertension, pre-eclampsia, and gestational diabetes.

### Breastfeeding information

Information on breastfeeding habits was obtained from well-baby clinics. All municipalities in Finland are obliged by law to organize a minimum of 15 preventive child care visits during the first 6 years of the child's life. Whether the child is breastfed is routinely recorded by healthcare providers at these visits. Breastfeeding information derived from the visit records was grouped in 2 variables: any breastfeeding, indicating whether the infant was ever breastfed or not; and breastfeeding at 6 months of age (with 0.5 months error margin), indicating whether the infant was breastfed at 6 months or not.

### Neighborhood characteristics

Characteristics of the living environment for each mother in the cohort were calculated based on residential addresses. Latitude and longitude coordinates and dates of all moves in the 5 years prior to child birth were obtained from the Population Register Centre. Using open-source Geographical Information Systems (QGIS; http://www.qgis.org), data on the residential neighborhoods were linked to the cohort participants’ home addresses by the latitude and longitude coordinates. Data for social living environment originated primarily from the Statistics Finland grid database, which contains socioeconomic information for Finnish residents at a spatial resolution of 250 × 250 m. These data include almost 100 key variables describing the structure of the population including level of education, median household income, unemployment rate, population density as well as buildings and workplaces within each map grid. Using the first 3 variables, we calculated a relative index of neighborhood SES for each grid (24).

The greenness variable was derived from multispectral satellite images (Landsat TM/OLI, 30 × 30 m of spatial resolution), which were used to calculate the normalized difference vegetation index (NDVI), as a measure of the green vegetation cover and density of plant growth (biomass) (25). Water bodies were masked out from the images and the NDVI values ranged from 0 to 1, where values close to 0 indicate areas with the lowest vegetation and values close to 1 indicate areas with the most dense vegetation. Neighborhood greenness was estimated as the mean of the NDVI within 250 × 250 m at the participants’ home addresses. NDVI is an unspecific measure of green vegetation presence. Different plant types, composition, and landscapes can have similar NDVI profiles because NDVI is not specific regarding green land cover types or their combinations (e.g., forests, grasslands, shrubs, mires). As a reference, low NDVI values can represent impervious asphalt-covered residential and industrial areas.

### Statistical analyses

Mean differences in neighborhood SES index, greenness index, and population density (continuous variables) across different classes of variables describing population characteristics were tested through ANOVA. Correlations between continuous descriptive variables and exposure variables were tested through Pearson correlations. For the purpose of the following analyses, both the neighborhood disadvantage score and the greenness score were divided into 3 categories. The neighborhood disadvantage score was classified based on national means: ≤ −0.5 (low disadvantage), from −0.5 to +0.5 (average disadvantage), and > +0.5 (high disadvantage). For greenness, a score ≤0.3 was categorized as low greenness, 0.3–0.6 as average greenness, and >0.6 as high greenness. The population density variable was used to derive a 2-category variable describing whether the neighborhood was highly populated [≥200 inhabitants/(250 × 250 m)] or scarcely populated [<200 inhabitants/(250 × 250 m)] (26). Differences in the distribution of any breastfeeding behavior (ever/never over the first 2 years of life) and at 6 months (breastfed/not breastfed) across different classes of the neighborhood disadvantage (low, average, high), greenness (low, average, high), and population density (scarcely populated, highly populated) variables were first checked through χ^2^ tests.

The presence of associations between the exposure to the neighborhood categorical variables (disadvantage, greenness, and population density) and the outcome variables (breastfed ever/never, breastfed at 6 months yes/no) were tested through unadjusted and adjusted binary logistic regression models. In this context, the 3 exposure variables were tested both in separate models and together in the same model, although separate models were preferred due to the correlations between the 3 exposure variables. Each adjusted regression model was adjusted for factors that were associated with breastfeeding practices in this cohort: individual SES variables (marital status, maternal occupation), maternal health (prepregnancy BMI, smoking during pregnancy, maternal disease diagnoses), infant and pregnancy characteristics (delivery mode, gestational age, sex, twin), and parity. Subgroups analyses, stratified by population density, were run in order to understand if the association of greenness and disadvantage with breastfeeding behavior was different in scarcely compared with highly populated settings. Sex-specific interactions were also tested by including the interaction term (e.g., exposure variable × infant sex) in each adjusted model. Similar models were tested with longer measurement intervals of cumulative disadvantage and greenness (≤15 y) with similar results, and they have not been reported in this manuscript. All statistical analyses were performed using IBM SPSS (version 25) and graphs were generated using Graph Pad Prism 8. The effects are expressed as adjusted ORs unless otherwise specified.

## Results

**Table 1** summarizes the background characteristics and primary outcomes in relation to the exposure variables for the study population (total *n* = 11,038). Mean maternal age in the study population was 29 ± 5 y, and the average BMI was 24.3 ± 4.8 kg/m^2^. Most mothers were healthy (81.4% had no chronic disease), and gave birth to singletons (98.5% of infants). Mean gestational age at birth was 39.8 ± 1.8 wk. Of the infants in the cohort, 97% were breastfed at some point in the first 2 years of life, but breastfeeding at 6 months was reported only for 60% of the infants. The correlation between each exposure variable is presented in **Supplemental Table 1. Supplemental Table 2** shows the distribution of individual SES across the different classes of each exposure variable.

**TABLE 1:** Descriptives of pre- and perinatal characteristics of the study population and their relation with the exposure variables (continuous)^1^

Maternal characteristics	Total *n* (%)	Disadvantage index^2^	*P* value	Greenness index^3^	*P* value	Population density [inhabitants/(250 × 250 m)]	*P* value
Age (y)	11,038 (100)	−0.24	<0.001	0.03	<0.001	−0.07	<0.001
Prepregnancy (BMI, kg/m^2^)	10,995 (91.5)	0.06	<0.001	0.10	<0.001	−0.01	<0.001
Gestational age (wk)	11,030 (99.9)	0.00	0.53	0.00	0.59	0.00	0.94
Previous births			0.008		<0.001		<0.001
None	5587 (50.6)	0.01 ± 0.56		0.49 ± 0.12		305 ± 231	
≥1	5451(49.4)	−0.02 ± 0.67		0.54 ± 0.10		225 ± 204	
Maternal socioeconomic status			<0.001		<0.001		<0.001
Higher-grade nonmanual	2352 (24.9)	−0.21 ± 0.51		0.49 ± 0.13		293 ± 242	
Lower-grade nonmanual	2199 (23.3)	−0.14 ± 0.53		0.52 ± 0.12		240 ± 205	
Manual	3187 (33.8)	0.07 ± 0.61		0.54 ± 0.11		230 ± 204	
Student	1214 (12.9)	0.12 ± 0.61		0.49 ± 0.12		328 ± 235	
Full-time mother	480 (5.1)	0.41 ± 0.76		0.52 ± 0.11		263 ± 204	
Birth mode			0.92		0.84		0.94
Vaginal	9502 (86.1)	−0.01 ± 0.62		0.51 ± 0.12		265 ± 221	
C-section	1536 (13.9)	−0.01 ± 0.61		0.51 ± 0.12		265 ± 221	
Maternal disease (any)			0.01		0.49		0.92
No	8981 (81.4)	−0.015 ± 0.61		0.51 ± 0.12		266 ± 222	
Yes	2057 (18.6)	0.03 ± 0.62		0.51 ± 0.12		265 ± 221	
Infant sex			0.85		0.39		0.29
Male	5406 (51.2)	−0.01 ± 0.61		0.51 ± 0.12		263 ± 218	
Female	5170 (48.8)	−0.01 ± 0.62		0.51 ± 0.12		268 ± 226	
Twin			0.96		0.81		0.68
No	10,872 (98.5)	−0.01 ± 0.61		0.51 ± 0.12		265 ± 222	
Yes	166 (1.5)	−0.01 ± 0.68		0.51 ± 0.11		272 ± 223	
Marital status			<0.001		0.003		<0.001
Married	6211 (56.3)	−0.06 ± 0.64		0.52 ± 0.12		258 ± 223	
Not married	4813 (43.7)	0.06 ± 0.57		0.50 ± 0.12		274 ± 220	
Smoking during pregnancy			<0.001		<0.001		0.70
No	9150 (83.1)	−0.06 ± 0.60		0.51 ± 0.12		266 ± 225	
Yes	1862 (16.9)	0.23 ± 0.60		0.52 ± 0.10		264 ± 206	
Any breastfeeding				<0.001			<0.001
Never	338 (3.1)	0.15 ± 0.67		0.56 ± 0.09	<0.001	165 ± 150	
Ever	10,700 (96.9)	−0.01 ± 0.61		0.52 ± 0.12		269 ± 223	
Breastfeeding at 6 months			0.01		<0.001		<0.001
No	4396 (39.8)	−0.03 ± 0.61		0.54 ± 0.12		197 ± 182	
Yes	6642 (60.2)	0.0 ± 0.62		0.49 ± 0.12		311 ± 234	

1 Values represent Pearson coefficients for continuous variables (i.e., maternal age, BMI, and gestational age) and mean ± SD of the exposure variables (i.e., neighborhood disadvantage index, neighborhood greenness index, and population density) for categorical factors. *P* values were obtained from ANOVA. ANOVA and Pearson correlation tests aimed at assessing associations and correlations between the categorical and continuous confounders and the continuous exposure variables. These variables have been calculated based on a spatial resolution grid of 250 × 250 m based on the residential neighborhood of the study participants.

2 Disadvantage index range: −2.377 to 4.050; higher values mean greater disadvantage.

3 Greenness index range: 0.1 to 0.8; higher values mean greater greenness.

**FIGURE 1:**
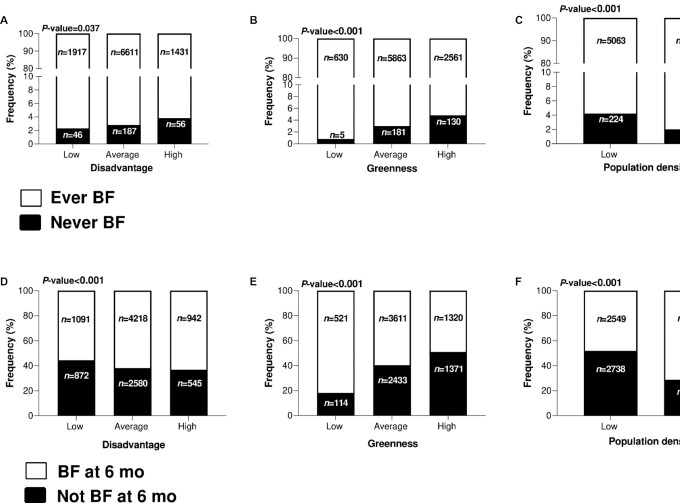
Distribution of any breastfeeding behavior and breastfeeding behavior at 6 months after birth across categories of neighborhood disadvantage (A and D), greenness (B and E), and population density (C and F). *P* values are obtained from Pearson χ^2^ tests and indicate general associations between the 2 categorical variables represented in each part of the figure. BF, breastfed.

The distribution of any breastfeeding behavior varied significantly across neighborhood socioeconomic disadvantage, greenness, and population density classes according to χ^2^ analyses: the percentage of never breastfed infants increased with disadvantage (3.8% in high disadvantage, compared with 2.8% and 2.3% in average and low disadvantage respectively; Figure 1A; *P* = 0.037) and greenness (4.8% in high greenness compared with 3.0% and 0.8% in average and low greenness, respectively; Figure 1B; *P* < 0.001) whereas it decreased with population density (2% in high-density neighborhoods compared with 4.2% in low-density ones; Figure 1C; *P* < 0.001), although >90% of infants were breastfed across all classes. Breastfeeding practices were associated with all exposure factors according to unadjusted logistic regression models (**Supplemental Table 3**).

Breastfeeding at 6 months across the different classes of socioeconomic disadvantage, greenness, and population density was also significantly different according to χ^2^ analyses (Figure 1D–F). In this context the greatest differences were observed across greenness classes, where 82% of the infants living in low greenness areas were breastfed at 6 months compared with 60% and 50% in the average and high greenness areas, respectively (Figure 1E; *P* < 0.001), and across population density classes, with 71% of infants from highly populated areas compared with 48% of infants from scarcely populated areas being breastfed at 6 months (Figure 1F; *P* < 0.001). Unadjusted logistic regression models showed similar results (**Supplemental Table 3**).

**FIGURE 2:**
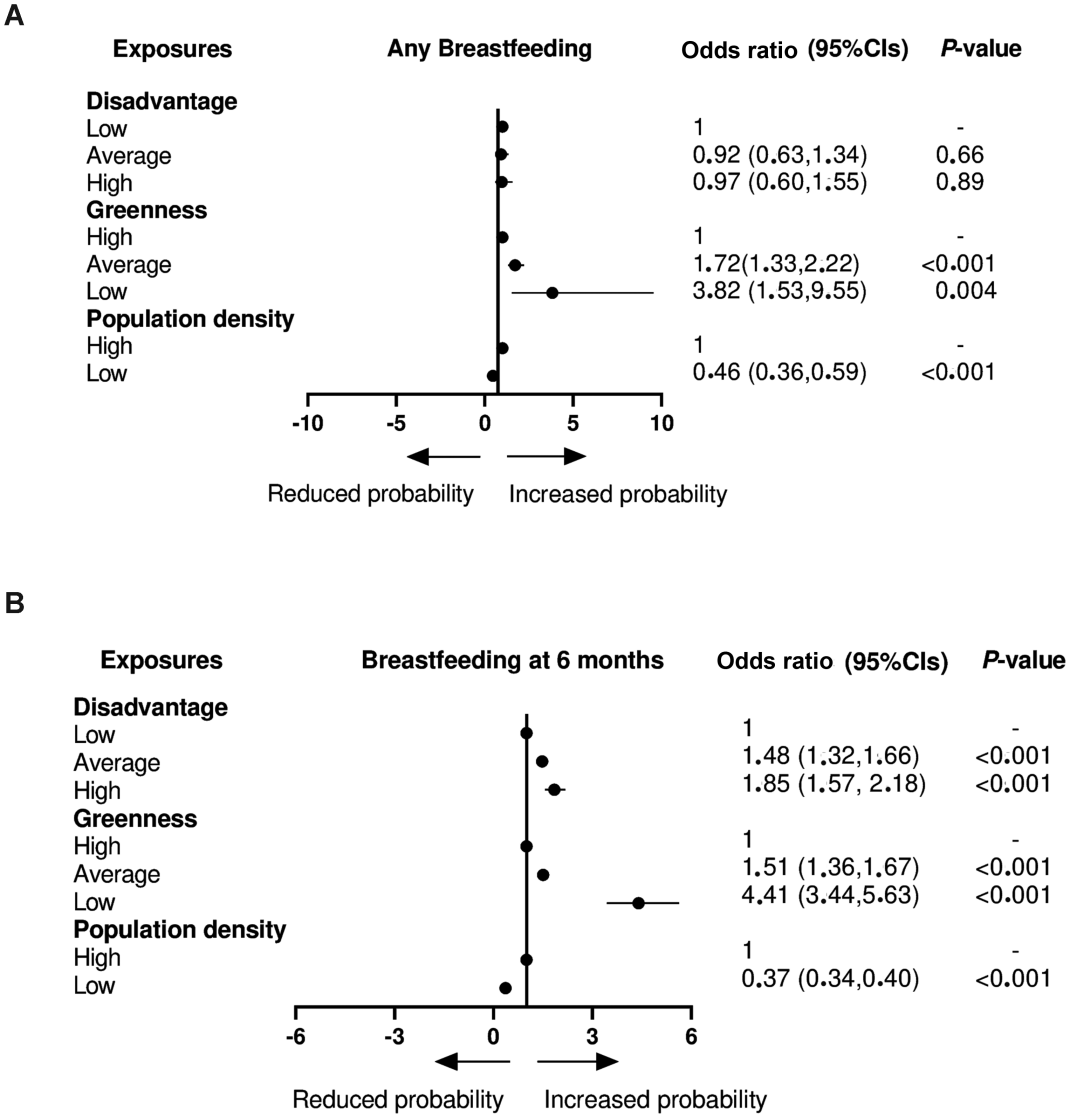
ORs for the any breastfeeding behavior (A, ever/never) and breastfeeding at 6 months (B, yes/no) in relation to exposure to living environment factors (neighborhood disadvantage, greenness, and population density). Logistic regression models were adjusted for the following confounders: infant's sex, maternal age, maternal prepregnancy BMI, parity, maternal occupational status, maternal diagnoses, gestational age, mode of delivery, marital status, and smoking during pregnancy. OR >1 indicates that infants are more likely to be breastfed compared with the reference category, and vice versa for OR <1. Neighborhood disadvantage score: ≤ −0.5 (low disadvantage), −0.5 to +0.5 (average disadvantage), and > +0.5 (high disadvantage). Greenness score: ≤0.3 (low greenness), 0.3–0.6 (average greenness), and >0.6 (high greenness). Population density: highly populated [≥200 inhabitants/(250 ×250 m)] and scarcely populated neighborhood [<200 inhabitants/(250 × 250 m)].

Adjusted logistic regression models confirmed the results gained from χ^2^ tests and unadjusted models, showing that greenness and population density were related to any breastfeeding, with mothers living in less green areas being more likely to breastfeed compared with mothers living in high greenness areas, and mothers living in highly populated areas having a greater probability to breastfeed at all compared with mothers living in scarcely populated areas (Figure 2A).

Adjusted models showed that neighborhood disadvantage, greenness, and population density were all significantly associated with breastfeeding at 6 months. In this context, mothers living in the most highly disadvantaged neighborhoods were more likely to breastfeed at 6 months compared with mothers living in the most affluent neighborhoods. Mothers living in less green and more populated areas were also more likely to breastfeed at 6 months compared with mothers living in the greenest areas and in the least populated neighborhoods (Figure 2B).

In subgroup analyses, where the total sample was divided by population density category, neighborhood disadvantage was associated with any breastfeeding behavior in mothers inhabiting scarcely populated areas (*P* = 0.05). Here more disadvantaged neighborhoods were linked to lower probability of breastfeeding at all compared with least disadvantaged neighborhoods (OR: 0.54; 95% CI: 0.30, 0.95). No statistically significant association between breastfeeding at all and disadvantage was observed in highly populated areas.

At 6 months, in subgroup adjusted analyses, where the total sample was divided by population density category, neighborhood greenness was significantly associated with breastfeeding behavior in mothers inhabiting highly populated areas (*P* < 0.001). Here mothers were more likely to breastfeed if they lived in neighborhoods with the least green spaces compared with mothers living in neighborhoods with the most green spaces (OR: 2.54; 95% CI: 1.74, 3.69). No similar effect was observed in scarcely populated areas (*P* > 0.05). Breastfeeding at 6 months was also linked to disadvantage in highly populated areas (*P* < 0.001), with mothers living in the most disadvantaged neighborhoods being more likely to breastfeed at 6 months compared with mothers living in the least disadvantaged neighborhoods (OR: 3.74; 95%CI: 1.92, 7.29). This association was not observed in scarcely populated areas.

## Discussion

Our results show that characteristics of the neighborhood in which the family lived up to 5 years before childbirth are linked to breastfeeding behavior. Therefore, the study provides an insight on the long-lasting role of neighborhood characteristics on breastfeeding behavioral patterns and suggests that more urbanized neighborhoods (e.g., less green and more populated) might be protective of breastfeeding.

Specifically, we observed that low neighborhood greenness and high population density were associated with higher probability to observe any breastfeeding and breastfeeding at 6 months. Low greenness was associated with higher breastfeeding probability, especially for mothers and children living in the most populated and less disadvantaged neighborhoods. Although it is well known that urban settings usually represent an increased opportunity to access healthcare services (21), the association between higher breastfeeding probability and low greenness in highly populated and most affluent neighborhoods was unexpected and contrary to our hypothesis, because previous studies have shown that maternal mental well-being is positively associated with breastfeeding behavior (27) and that the presence of green areas in the neighborhood positively affects mental well-being of the inhabitants (22).

Neighborhood disadvantage in the present study was significantly associated with breastfeeding at 6 months in the whole cohort and in highly populated areas, and with any breastfeeding in mothers inhabiting scarcely populated areas. In this context we found that, cohort-wide, mothers living in more disadvantaged settings were more likely to breastfeed at 6 months compared with mothers living in less disadvantaged settings. This was shown to be especially significant for mothers living in highly populated neighborhoods. In scarcely populated neighborhoods we instead observed that mothers living in more disadvantaged settings were less likely to breastfeed at all compared with mothers in least disadvantaged neighborhoods.

The observation that mothers would more likely breastfeed at 6 months if inhabiting more disadvantaged settings in highly populated areas appears to contrast with previous literature, which supports the idea that low neighborhood purchasing power is linked to early cessation of breastfeeding (18) and that breastfeeding is economically more costly to mothers than formula feeding (28). However, according to Finnish legislation, over 4 months of paid parental leave is provided for mothers, and further parental allowance to support childcare is also universally available. This might encourage more disadvantaged mothers to take extra parental leave and breastfeed their infant for longer, as opposed to resuming their full-time work schedule and choosing to formula-feed (29). The pattern might instead be reversed for mothers in higher occupational positions. Yet, this is merely speculation because, although we did correct for mothers' occupational level, we did not have access to data on parental leave and other benefits for this cohort. Further analyses of individual SES factors in relation to neighborhood SES, governmental resources and use, and reasons for breastfeeding cessation are needed for a deeper insight on this matter. Additionally, some studies show that the amount of support that mothers receive from fathers can also influence infant feeding choices (30). Future studies should investigate the associations between paternal support and breastfeeding rates in relation to neighborhoods SES.

Although our data do not support an explanation of why more disadvantaged neighborhoods in highly populated areas seem to have a protective effect on breastfeeding, the difference between scarcely populated and highly populated areas is very interesting and consistent with the hypothesis that even in wealthy countries such as Finland, families inhabiting scarcely populated areas might be disadvantaged in terms of healthcare support and/or education. This could be especially true if the family lives in a neighborhood with low SES. In highly populated areas, low SES does not seem to negatively affect breastfeeding.

The main strength of the present study is the large sample size of the unselected, population-based cohort and the high attendance of mothers to well-baby clinic follow-up. Furthermore, another major strength of our study was the utilization of a high-resolution (250 × 250 m) grid database linked to data from all residential addresses of the study participants over a period of 5 years until childbirth. These strengths combined allowed an accurate assessment of neighborhood characteristics on breastfeeding practices in the population of southwest Finland, although ∼16% of the original birth cohort was excluded from this study because it was missing breastfeeding information. In this context, adherence to the visits at municipal clinics (where breastfeeding is collected) is generally high, and the proportion of those not attending any visits has been estimated to be as low as 0.5% based on vaccination coverage. A small proportion of the missing data can be explained by the study subjects moving to geographical areas outside of southwest Finland. Thus, the likely explanation for most missing breastfeeding data is related to gaps in data acquisition from municipalities using different electronic record systems. Finally, it is possible that recording of breastfeeding information varied or was inaccurate in some communities. Yet, the present study was to our knowledge the first to comprehensively analyze the association between long-term (5 y) environmental exposures and breastfeeding practices using detailed information on neighborhood disadvantage, geographical characteristics, and population density. Unfortunately, due to the lack of detailed information around individual SES, our analyses could not be adjusted for factors such as education and household income. However, we did correct the analyses for maternal occupational level, which is strongly related to education and income. Moreover, in this study we did not control for immigration status although it is possible that immigrants have different breastfeeding patterns and are more likely to live in lower SES urban settings. However, only 6% of the mothers were immigrants in this birth cohort (31), thus a major bias from immigration status is unlikely. Additionally we were not able to include information on smoking after pregnancy as a confounder in our models. This factor could potentially confound the results because the rate of smoking is likely to increase after birth compared with that observed in pregnancy. However, this information was unfortunately not collected. Another weakness of the present study was the availability of only general breastfeeding outcomes. However, although the study could have benefitted from further information such as duration of exclusive breastfeeding, supplementation with formula feeding, total duration of breastfeeding, and reason for breastfeeding cessation, it is also practically very difficult to obtain such specific information from a large cohort such as the one used in this study.

Overall, the cost that breastfeeding entails for mothers, paired with adverse or inappropriate environmental exposures, could impact on breastfeeding behavior. The resources and characteristics of the neighborhoods might contribute to whether and for how long mothers breastfeed their infants. The present study suggests that this might be the case even in countries like Finland, where social welfare and education are well developed and seemingly accessible to everyone at similar levels. The presence of marginalized and disadvantaged neighborhoods within societies that are thought of as uniformly wealthy should be acknowledged and acted on. The present study reveals that the combination of high neighborhood disadvantage, high greenness, and low population density of family neighborhoods in southwest Finland shortens breastfeeding practices and therefore might contribute to the establishment of early life health inequalities for babies that are born in these settings. Future interventional studies should aim at understanding what action can be taken to ensure that the different environments where families live have equal resources to support mothers who intend to breastfeed. These resources might include community-based interventions highlighting the importance of prolonged breastfeeding for newborns, and improved family and societal childcare support. Breastfeeding is an essential part of maternal and infant health, and global efforts are being made to highlight the right of every infant to be breastfed according to guidelines. Therefore, the acquisition of knowledge around specific neighborhood and environmental factors that might affect the breastfeeding likelihood for children in different settings worldwide is essential. This will allow us to better channel efforts aimed at reducing health inequality and early life disparities.

## Supplementary Material

nxac069_Supplemental_File

## Data Availability

The dataset supporting the conclusions of this article can be made available upon request to the corresponding author after approval is obtained from the STEPS Study Executive Committee.

## Notes

This study was supported by the Academy of Finland (32409 to HL, 329240 to JV and 329202 to MK), the Juho Vainio Foundation (202010259 to HL) and Finnish Work Environment Fund, Finland (190424 to MK) and Special Governmental grants for Health Sciences Research (Turku University Hospital).

Author disclosures: SR received the following: Honorarium for lecture from Nutricia and Honoraria for lectures and contribution to a textbook from the Nestlé Nutrition Institute. All other authors report no conflicts of interest.

Supplemental Figure 1 and Supplemental Tables 1–3 are available from the “Supplementary data” link in the online posting of the article and from the same link in the online table of contents at https://academic.oup.com/jn/.
