# Supplementary material for: Neighborhood Disadvantage, Greenness, and Population Density as Predictors of Breastfeeding Practices: A Population Cohort Study from Finland
Source: J Nutr. 2022 Mar 24;152(7):1721–8. doi: 10.1093/jn/nxac069 (PMC9258599; doi:10.1093/jn/nxac069)
Supplement: nxac069_Supplemental_File [file nxac069_supplemental_file.docx]

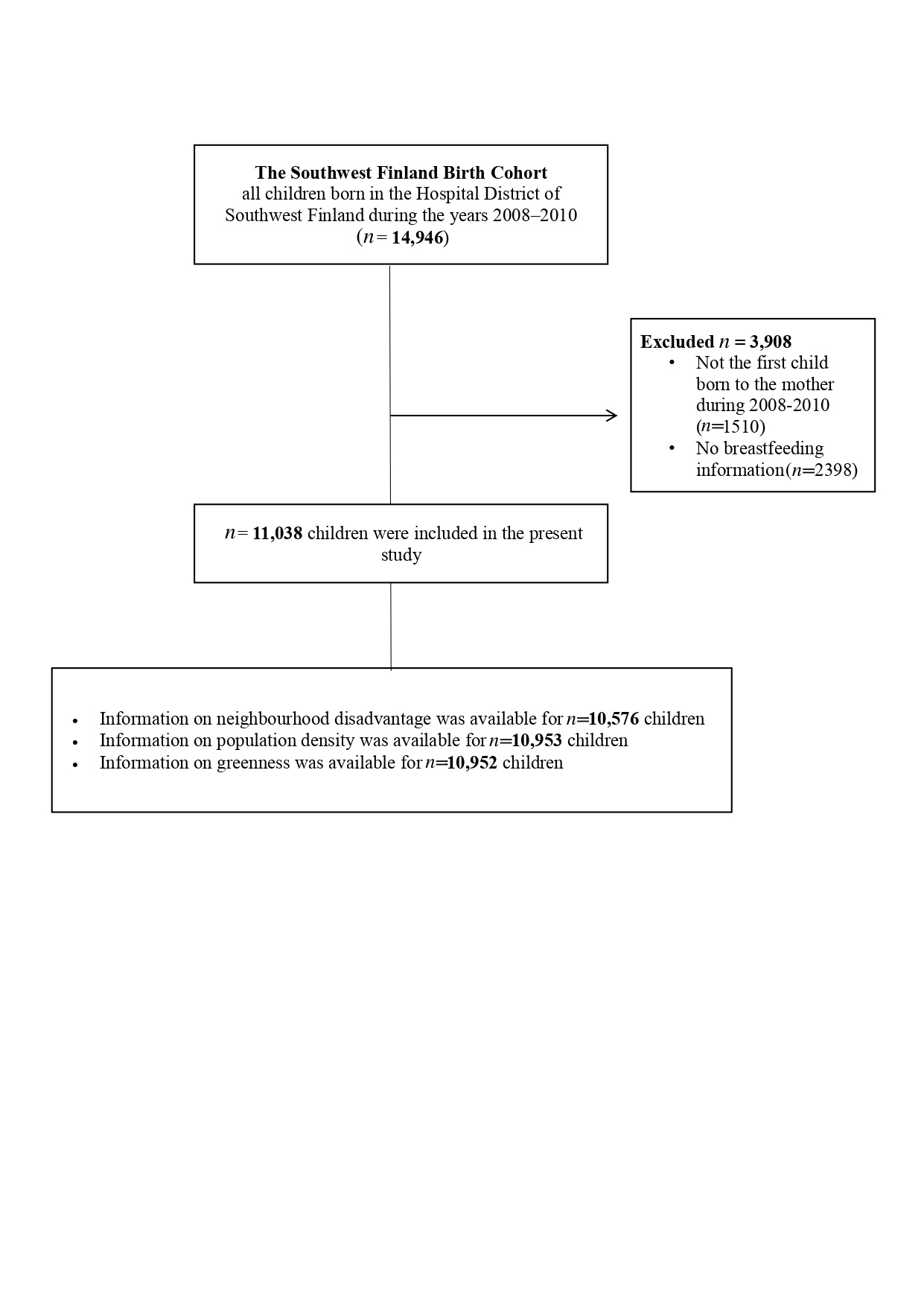


**Supplementary Figure 1**. Flowchart of the inclusion criteria for study population.

***Supplementary Table 1.*** *Pearson correlation across the three exposure variables (continuous)*

|  | **Disadvantage** | | **Population density** | | **Greenness** | |
| --- | --- | --- | --- | --- | --- | --- |
|  | **Pearson coefficient** | ***P*-value** | **Pearson coefficient** | ***P*-value** | **Pearson coefficient** | ***P*-value** |
| **Disadvantage** | 1 | - | 0.172 | <0.001 | -0.105 | <0.001 |
| **Population density** | 0.172 | <0.001 | 1 | - | -0.738 | <0.001 |
| **Greenness** | -0.105 | <0.001 | -0.738 | <0.001 | 1 | - |

***Supplementary Table 2.*** *Distribution of Individual maternal SES across each exposure variable.*

| **Mother's Occupation^a^** | **High disadvantage** | **Average disadvantage** | **Low disadvantage** |
| --- | --- | --- | --- |
|  | ***n* (%)** | ***n* (%)** | ***n* (%)** |
| Higher-grade non-manual | 109 (9.2) | 1545 (26.3) | 562 (32.9) |
| Lower-grade non-manual | 160 (13.5) | 1385 (23.6) | 504 (29.5) |
| Manual | 533 (45.0) | 1907 (32.5) | 473 (27.7) |
| Student | 220 (18.6) | 812 (13.8) | 125 (7.3) |
| Full-time mother | 162 (13.7) | 215 (3.7) | 43 (2.5) |
| **Mother's Occupation^a^** | **High greenness** | **Average greenness** | **Low greenness** |
|  | ***n* (%)** | ***n* (%)** | ***n* (%)** |
| Higher-grade non-manual | 506 (21.7) | 1206 (23.4) | 236 (44) |
| Lower-grade non-manual | 554 (23.8) | 1223 (23.7) | 114 (21.3) |
| Manual | 945 (40.6) | 1781 (34.6) | 96 (17.9) |
| Student | 209 (9) | 675 (13.1) | 83 (15.5) |
| Full-time mother | 115 (4.9) | 268 (5.2) | 7 (1.3) |
| **Mother's Occupation^a^** | **High Population density** | **Low population density** | **-** |
|  | ***n* (%)** | ***n* (%)** | ***-*** |
| Higher-grade non-manual | 1296 (27.3) | 1033 (22.5) | ***-*** |
| Lower-grade non-manual | 998 (21.0) | 1190 (25.9) | ***-*** |
| Manual | 1417 (29.8) | 1745 (37.9) | ***-*** |
| Student | 787 (16.6) | 416 (9) | ***-*** |
| Full-time mother | 251 (5.3) | 217 (4.7) | ***-*** |

^a^Individual SES information were collected before birth.

***Supplementary Table 3.*** *Unadjusted odd ratios for breastfeeding behaviour in relation to single exposure variables.*

|  | **Any breastfeeding** | | **Breastfeeding at 6 months** | |
| --- | --- | --- | --- | --- |
|  | **Unadjusted Odd ratio (95% CI)** | ***P*-value** | **Unadjusted Odd ratio (95% CI)** | ***P*-value** |
| Disadvantage^1^ |  |  |  |  |
| Low | Reference | - | Reference | - |
| Average | 0.85 (0.61,1.18) | 0.32 | 1.31 (1.18, 1.45) | <0.001 |
| High | 0.61 (0.41, 0.91) | 0.02 | 1.38 (1.20, 1.59) | <0.001 |
| Greenness |  |  |  |  |
| High | Reference | - | Reference |  |
| Average | 1.64 (1.31, 2.07) | <0.001 | 1.54 (1.41,1.69) | <0.001 |
| Low | 6.40 (2.61, 15.69) | <0.001 | 4.75 (3.82, 5.89) | <0.001 |
| Population density |  |  |  |  |
| High | Reference | - | Reference | - |
| Low | 0.45 (0.36, 0.57) | <0.001 | 0.38 (0.35, 0.41) | <0.001 |

^1^standardized z-score based on the total Finnish population

OR>1 indicates that infants are more likely to be breastfed compared to the reference category and vice versa for OR<1*.*

Neighborhood disadvantage score: ≤ -0·5 (low disadvantage), from -0·5 to +0·5 (average disadvantage) and > +0·5 (high disadvantage).

Greenness score: low, NDVI ≤ 0·3; average, NDVI 0·3-0·6; high, NDVI>0·6 .

Highly populated, ≥200 inhabitants/(250m x 250m); scarcely populated, <200 inhabitants/(250m x 250m).
